# Health and Recreation

**Published:** 1920-01-31

**Authors:** 


### Health and Recreation.

It was not a very cheerful survey which Dr. Herbert Carson delivered at the Institute of Hygiene on "Amusements and their Effects." There is a definite ratio, he says, between efficiency and fatigue, so that a shorter working day might result in greater output, better work, and fewer accidents, but the consequent increase of leisure makes it necessary that the workers should be taught how to spend it. He considers the workers should be instructed in the value of leisure—at any rate the value of the proper use of leisure, which is what he seems to mean. For his review of present-day use of leisure is not very favourable.

Rural workers, tired of the fields, resort to the village inn for gossip, and ought to be provided with clubs, reading-rooms, libraries, discussion circles, and social games. One fears, however, that that much resembles the introduction of the horse to the stream without being able to compel him to drink, and the same remark applies to Mr. Carson's references to the cheap and unhealthy cinemas, theatre revues, spectators at professional-football matches, night clubs and bridge clubs—"becoming a serious scandal"—and the use of public-houses (which, he declares, are a disgrace to our civilisation) as the poor man's clubs. It is a commonplace that active amusements provide the ideal way of spending leisure, but cinemas, theatres, and public-houses are perfectly legitimate forms of social leisure; and what is required is the education of people to better tastes, together with insistence upon healthy buildings so far as possible—both big undertakings.

Mr. Carson thinks it is time the allotment plots were returned for the use of children's games, and the question of the amusements of children he regards as the most important aspect of the whole subject. He is undoubtedly on firm ground in giving this latter point prominence, for in the education and training of children in games, as in most things, lies the most hopeful line of progress.

It is good to know that great advance is taking place in active recreation for women. The games mistress and director of physical education is now an important member of the staff at all schools; and the girl of to-day, with her well-developed body and fearless outlook on life, compares most favourably with her Victorian predecessor. The social-welfare movement among working women instils in them the general principles of hygiene, improves their health, and makes their work a pleasure to them. Little has yet been done for the working man's wife. She has increased leisure now that children can be fed at the schools and entertained at play centres, but little recreation has been provided for her.

Mr. Carson is not much perturbed by the craze for dancing, from which he sees the production of much useful development when tendencies to abuse have adjusted themselves. He has a gentle word of warning for the hard-driven business man who rushes off on Saturday for an orgy of week-end exercise. A sensible mind in a healthy body is necessary for a healthy mind in a healthy body. Abuse of either can only be indulged in at a price.

